# Creation and implementation of a European registry for patients with McArdle disease and other muscle glycogenoses (EUROMAC registry)

**DOI:** 10.1186/s13023-020-01455-z

**Published:** 2020-10-15

**Authors:** Tomàs Pinós, Antoni L. Andreu, Claudio Bruno, Georgios M. Hadjigeorgiou, Ronald G. Haller, Pascal Laforêt, Alejandro Lucía, Miguel A. Martín, Andrea Martinuzzi, Carmen Navarro, Piraye Oflazer, Jean Pouget, Ros Quinlivan, Sabrina Sacconi, Renata S. Scalco, Antonio Toscano, John Vissing, Matthias Vorgerd, Andrew Wakelin, Ramon Martí, EUROMAC Consortium, Antoni L. Andreu, Ramon Martí, Tomàs Pinós, Noemi Baruch, Francisco J. Ortega, Miguel A. Martín, Carmen Navarro, Beatriz San-Millán, Irene Vieitez, Andrea Martinuzzi, Marinela Vavla, Claudio Bruno, Antonio Toscano, Olimpia Musumeci, Pascal Laforêt, Sabrina Sacconi, Ros Quinlivan, Renata Scalco, Andrew Wakelin, Georgios Hadjgeorgiou, Elias Zintzaras, John Vissing, Matthias Vorgerd, Enrico Zülow, Ronald Haller, Piraye Oflazer, Hacer Durmus, Jean Pouget, Alejandro Lucía, Alfredo Santalla

**Affiliations:** 1 Biomedical Network Research Centre on Rare Diseases (CIBERER), Instituto de Salud Carlos III, and Research Group on Neuromuscular and Mitochondrial Diseases, Vall d’Hebron Research Institute, Universitat Autònoma de Barcelona, Pg. Vall d’Hebron 119, 08035 Barcelona, Catalonia Spain; 2 Center of Translational and Experimental Myology, IRCCS Istituto Giannina Gaslini, Genoa, Italy; 3 Department of Neurology, Laboratory of Neurogenetics, University of Thessaly, Volos, Greece; 4 Neuromuscular Center, Institute for Exercise and Environmental Medicine of Texas Health Presbyterian Hospital, Dallas, TX USA; 5 Nord/Est/Ile de France Neuromuscular Reference Center, Neurology Department, Raymond-Poincaré Teaching Hospital, AP-HP, Garches, France; 6 INSERM U1179, END-ICAP, Paris Saclay University, Paris, France; 7 Faculty of Sport Sciences, Universidad Europea de Madrid, Madrid, Spain; 8 Biomedical Network Research Centre on Rare Diseases (CIBERER), Instituto de Salud Carlos III, and 12 de Octubre University Hospital Research Institute, (‘imas12’), Madrid, Spain; 9 Department of Conegliano-Pieve di Soligo, IRCCS Eugenio Medea-Associazione “La Nostra Famiglia” Scientific Institute, Bosisio Parini, Italy; 10 Institute of Biomedical Research of Vigo, Vigo, Spain; 11 Department of Neurology, Neuromuscular Unit, Istanbul University, Istanbul, Turkey; 12 Centre de Référence Maladies Neuromusculaires, Assistance Publique-Hopitaux de Marseille, Marseille, France; 13 MRC Centre for Neuromuscular Diseases, UCL Institute of Neurology, National Hospital, London, UK; 14 University of Nice, Nice, France; 15 Neurology and Neuromuscular Diseases Unit, Department of Clinical and Experimental Medicine, University of Messina, Messina, Italy; 16 Rigshospitalet, University of Copenhagen, Copenhagen, Denmark; 17 Heimer Institute for Muscle Research, University Hospital Bergmannsheil Bochum, Bochum, Germany; 18 Association for Glycogen storage Disease, Bristol, UK

**Keywords:** Myopathy, Rare diseases, International registry, McArdle disease, Metabolic diseases, Glycogen storage disease

## Abstract

**Background:**

International patient registries are of particular importance for rare disorders, as they may contribute to overcome the lack of knowledge derived from low number of patients and limited awareness of these diseases, and help to learn more about their geographical or population-based specificities, which is relevant for research purposes and for promoting better standards of care and diagnosis. Our objective was to create and implement a European registry for patients with McArdle disease and other muscle glycogenoses (EUROMAC) and to disseminate the knowledge of these disorders.

**Results:**

Teams from nine different countries (United Kingdom, Spain, Italy, France, Germany, Denmark, Greece, Turkey and USA) created a consortium that developed the first European registry dedicated to rare muscle glycogenoses. A work plan was implemented to design the database and platform that constitute the registry, by choosing clinical, genetics and molecular variables of interest, based on experience gained from previous national registries for similar metabolic disorders. Among dissemination activities, several teaching events were organized in different countries, especially those where the consortium considered the awareness of these diseases needs to be promoted among health professionals and patients.

**Conclusion:**

EUROMAC represents a step forward in the knowledge of those disorders to which it is dedicated, and will have relevant clinical outcomes at the diagnostic, epidemiological, clinical and research level.

## Background

McArdle disease (glycogen storage disease type V, myophosphorylase deficiency) is an autosomal recessive disorder of glycogen metabolism, originally described in 1951 [1]. It is the most frequent metabolic myopathy with an estimated prevalence of ~ 1 in 140,000 people [2]. The disease is caused by mutations in *PYGM* that encodes the muscle isoform of glycogen phosphorylase (GP-MM; 1,4-alpha-D-glucan: phosphate alpha-D-glucosyltransferase). The mutations lead to loss of enzyme activity and thus inability to break down muscle glycogen, making this source of energy unavailable to patients [3]. Clinically, McArdle Disease is characterized by exercise intolerance typified by acute crises of early fatigue and muscle contractures that in ~ 50% of the cases are accompanied by rhabdomyolysis, hyper-creatine kinase (CK)-emia and myoglobinuria, which occasionally result in acute renal failure [3, 4]. In case of delayed diagnosis and incorrect medical advice, this could lead to risk of life-threatening crises necessitating admission to critical care. There is phenotypic variability, ranging from rare cases of patients that are virtually asymptomatic to the more common ones reporting recurrent exertional myoglobinuria, fixed muscle weakness and severe limitations of exercise and most daily life activities [2, 5].

There are scarce data available on the McArdle Disease epidemiology, as most of the studies have been limited by small sample sizes, consistently < 100 cases [4, 6–20]. The study of large cohorts of patients with rare disorders such as McArdle Disease is hampered by the low prevalence and heterogeneity of patient populations, as well as by the limited knowledge of their natural history. The largest cohort of McArdle Disease patients was reported in 2012, which described the features of 239 Spanish patients [5], updated 5 years later with 94 additional patients [2]. Studies of larger cohorts are clinically important as they help to improve our knowledge about the disease and of the main problems faced by patients, thereby allowing for targeted lifestyle intervention. Thus, the European Commission and the National Institutes of Health have set-up international patient registries for rare diseases to facilitate knowledge about them and to promote better standards of care and diagnosis.

We aimed to implement a European Patient Registry for McArdle Disease and other rare muscle glycogenoses (EUROMAC registry, EMR). The present paper describes how the EMR was developed and implemented detailing its strengths and shortcomings with the aim of facilitating the creation of other rare disease patient registries.

## Methods and results

### Aims of the EMR

The main aim of the EMR was to promote awareness and understanding of McArdle Disease and other rare muscle glycogenoses (types 0, III, IV, VII, VIII, IX, X, XI, XII, XIII, XIV and XV). Glycogenosis type II (Pompe disease) was not included in EUROMAC because a global registry for Pompe disease already exists (NCT00231400, https://clinicaltrials.gov/), which is planned to last at least until year 2034. To achieve this main goal, several specific aims were proposed: a) to obtain epidemiology data on these diseases in Europe; b) to reach patients and medical community to inform on these disorders; c) to assess clinical and cost effectiveness of standard and novel therapeutic interventions; d) to assess quality of care provided by services; e) to gain insight on disease pathophysiology; and f) to provide an inventory of patients that can be contacted for clinical research. In pursuance of these objectives, the EMR intended to recruit into the registry all individuals who had been diagnosed with McArdle Disease and other rare muscle glycogenoses in Europe. Thus, the expected long-term impacts of the EMR are as followed: 1) an improved access by patients to specialized care; 2) facilitation of the involvement of governments and regulatory agencies; 3) improved knowledge of the natural history of these diseases; 4) shorter delay in diagnosis; 5) reduced risk of incorrect advice leading to debilitating symptoms and risk of life-threatening crises, and 6) extended use of the registry to all European countries. The possibility to increase the expertise on these disorders in eastern European countries makes this goal especially valuable.

### Target groups

EUROMAC has promoted the involvement of a number of stakeholders, such as:

- Patients and their relatives, as well as caregivers.
- Patients’ advocacy groups (e.g., patients’ associations).
- Clinicians and researchers, participating as data contributors.
- Biopharmaceutical industry.
- Governments and regulatory agencies.

### Implementation

EUROMAC was founded by joining 13 full partners from seven EU member countries at the time of the consortium building (United Kingdom, Spain, Italy, France, Germany, Denmark and Greece) plus five collaborative members from four countries (Spain, France, Turkey and USA). The initial building of the consortium aimed to apply for funding to the European Commission’s Consumers, Health, Agriculture and Food Executive Agency (CHAFEA) to develop the EUROMAC project (see “Funding and Acknowledgements” section below) and so partners were divided into two categories according to the specificities of the CHAFEA call: full partners were in charge of specific tasks, receiving funds budgeted for those tasks, while collaborative partners provided expertise and valuable collaboration, but did not receive direct funding as they were not responsible of specific tasks. Travel expenses for EUROMAC meetings and events were covered for both full and collaborative partners. The complete list of members is shown in Table 1. The EUROMAC team constituted a multidisciplinary group in the field of neuromuscular disorders including neurologists, paediatricians, physiotherapists, exercise physiologists, clinical psychologists, biologists, patients’ representatives as well as project and administrative managers. Additionally, EUROMAC benefited from the experience gained by the different partners in obtaining their own patient cohorts and national registries [2, 4–6, 12, 19, 20].

**Table 1:** List of partners and collaborative members of the EUROMAC consortium

Membership	Organization name	Acronym	Department	Type of partner	City	Country
Full partner	Vall d’Hebron Institut de Recerca	VHIR	Neuromuscular and Mitochondrial Research	Academic/Hospital	Barcelona	Spain
Full partner	Servicio Madrileño de la Salud	SERMAS	Mitochondrial and Neuromuscular Diseases group	Academic/Hospital	Madrid	Spain
Full partner	Fundación Biomédica del Complejo Hospitalario Universitario de Vigo	FBCHUVI	Pathology and Neuropathology	Academic/Hospital	Vigo	Spain
Full partner	IRCCS Eugenio Medea-Associazione “La Nostra Famiglia” Scientific Institute	MEDEA	Conegliano-Pieve di Soligo Research Centre	Academic/Hospital	Conegliano	Italy
Full partner	IRCSS Giannina Gaslini	IGG	Neuroscience	Academic/Hospital	Genova	Italy
Full partner	University of Messina	UMESSINA	Neurology and Neuromuscular diseases Unit, Department of Clinical and Experimental Medicine.	Academic/Hospital	Messina	Italy
Full partner	Assistance Publique-Hopitaux de Paris	APHP	Clinical Research and Development/Institut de Myologie	Academic/Hospital	Paris	France
Full partner	Centre Hospitalo-Universitaire de Nice	CUH-NICE	Centre de Référence Maladies Neuromusculaires et SDA	Academic/Hospital	Nice	France
Full partner	Institute of Neurology, University College of London	UCL-MRC	MRC Centre for Neuromuscular Disease	Academic/Hospital	London	UK
Full partner	Association for Glycogen Storage Disease (UK)	AGSD-UK	Not applicable	Patient organization	Droxford	UK
Full partner	Faculty of Medicine -School of Health Sciences-University of Thessaly	UTH	Neurology	Academic/Hospital	Larissa	Greece
Full partner	Rigshospitalet, University of Copenhagen	RIGUC	Neurology	Academic/Hospital	Copenhagen	Denmark
Full partner	Berufsgenossenschaftliches Universitätsklinikum Bergmannsheil GmbH	BGH	Neuroscience	Academic/Hospital	Bochum	Germany
Collaborative partner	Neuromuscular Center, Institute for Exercise and Environmental Medicine	IEEM	Neuromuscular Disorders	Academic/Hospital	Dallas	USA
Collaborative partner	Istanbul University	IU	Neurology	Academic/Hospital	Istambul	Turkey
Collaborative partner	Assistance Publique-Hopitaux de Marseille	AP-HM	Centre de Référence Maladies Neuromusculaires	Academic/Hospital	Marseille	France
Collaborative partner	Universidad Europea de Madrid	UEM	Not applicable	Academic	Madrid	Spain
Collaborative partner	Instituto de Salud Carlos III	ISCIII	Not applicable	Health Research Agency	Madrid	Spain

Initially, the registry was restricted to European countries, as the project was framed within a funding contract with the CHAFEA agency of the European Commission. However, the aim to expand the registry to non-European countries in the future was also stated since the beginning. EUROMAC is now open to accept new professionals from non-European countries, provided they follow the same procedures already applied to the existing centres (namely, translation of the patient information and consent form to the local language, and approval by the local Ethics committee of centre to which the new participant form an external country belongs).

All EUROMAC actions were supported and funded by the agencies quoted at the “Funding and Acknowledgements” section, at the end of this article. The implementation of the EUROMAC project was divided into eight work packages (WP) as detailed in Table 2. A Steering Committee (constituted by the eight WP leaders) was established to provide guidance on the development, implementation and management of the project. Additionally, the Steering Committee facilitated the design, content and structure of the registry utilizing the varied skill sets of individual members. All decisions were taken during 10 different meetings (five exclusively for the Steering Committee and the remainder with the participation of members of the whole consortium) that took place in Luxembourg, London, Brighton, Conegliano, Roma, Madrid, Villaviciosa de Odón and Barcelona, between May 2013 (kick-off meeting, Luxembourg) and July 2016 (final project meeting, Barcelona). Other work meetings to discuss and manage specific tasks related with the implementation of the registry took place as needed.

**Table 2:** List of work packages and their corresponding leading centre in the EUROMAC consortium

		Leading Centre
**Horizontal Work Packages**		
WP1	Coordination	VHIR
WP2	Dissemination	AGSD-UK
WP3	Evaluation	MEDEA
**Core Work Packages**		
WP4	Methodology and registering activity	SERMAS
WP5	Quality assessment and management	UTH
WP6	Data analysis	RIGUC
WP7	Training and education	UCL-MRC
WP8	Ethical and legal issues	APHP

### Structure of the registry

The data obtained from patients and included in the registry were divided in eight different items: 1) demographic data; 2) diagnostic data; 3) clinical data; 4) data on concomitant diseases; 5) genetic data; 6) self-reported functional data; 7) previous and/or ongoing treatments and 8) services provided. The full list of variables introduced in the registry is shown in Table 3.

**Table 3:** List of variables included in the EUROMAC registry applicative^a^

	Type of field	Optional/Required field	Field accessible by patients
**Personal data**^**b**^			
Firs name	To fill	Required	Yes
Last name	To fill	Required	Yes
Email address	To fill	Optional	Yes
Date of birth	Date	Required	Yes
Phone number	To fill	Optional	Yes
City of birth	To fill	Required	Yes
Country of birth	Dropdown menu	Required	Yes
City of residence	To fill	Optional	Yes
Country of residence	Dropdown menu	Optional	Yes
Sex	Male/Female choice	Required	Yes
Height (cm)	To fill	Optional	Yes
Weight (kg)	To fill	Optional	Yes
**1.Demographic data**^**c**^			
Case code	Automatically generated	Required	No
Age	Generated from personal data	Required	Yes
Country of birth	Generated from personal data	Required	Yes
Sex	Generated from personal data	Required	Yes
Height	Generated from personal data	Optional	Yes
Weight	Generated from personal data	Optional	Yes
Body mass index	Generated from personal data	Optional	Yes
**2.Diagnosis**			
First diagnostic centre	Dropdown menu	Required	No
Diagnosing age	To fill	Required	No
Diagnosed disease	Dropdown menu	Required	No
Diagnosing through biopsy	Yes/No choice	Required	No
Diagnosing through genetic testing	Yes/No choice	Required	No
Genetic mutations	Allele 1 Dropdown menu	Required	No
	Allele 2 Dropdown menu	Required	No
Reference sequence used	To fill	Optional	No
**3.Clinical data**			
Second wind	Yes/No/NA choice	Required	Yes
Myoglobinuria	Yes/No/NA choice	Required	Yes
Systems with disease (affected organs)	Dropdown menu	Required	No
Difficulties in maintaining physical activities	Yes/No choice	Required	No
Weakness	Yes/No choice	Required	No
Muscle wasting	Yes/No choice	Required	No
Disease severity	Dropdown menu	Optional	No
**Lab data**			
Forearm test	Performed/Not performed	Required	No
Basal serum CK levels	To fill	Optional	No
Other tests (specify)	To fill	Optional	No
**Exercise testing**			
VO_2_ peak	To fill	Optional	No
%HRmax predicted	To fill	Optional	No
VO2 peak power output	To fill	Optional	No
Lab demonstrated second wind	To fill	Optional	No
Other tests (specify)	To fill	Optional	No
**4.Concomitant diseases**			
Diabetes	Yes/No/NA choice	Optional	No
Coronary Artery Disease	Yes/No/NA choice	Optional	No
Hypertension	Yes/No/NA choice	Optional	No
Cancer	Yes/No/NA choice	Optional	No
COPD	Yes/No/NA choice	Optional	No
Acute renal failure	Yes/No/NA choice	Optional	No
Chronic renal failure	Yes/No/NA choice	Optional	No
Hyperuricemia or Gout	Yes/No/NA choice	Optional	No
Anemia or Hyperbilirubinemia	Yes/No/NA choice	Optional	No
Other comorbidity	To fill	Optional	No
**5.Other genetic factors**			
ACE Genotype	I/I, I/D, D/D or N/D choice	Optional	No
ACTN3 Genotype	R/R, R/X, X/X or N/D choice	Optional	No
AMPD1 Genotype	Q/Q, Q/X, X/X or N/D choice	Optional	No
PGC Genotype	G/G, G/S, S/S or N/D choice	Optional	No
Other Genotype	To fill	Optional	No
**6.Self-reported functional data**			
Limitations	Yes/No/NA choice	Optional	Yes
WHO DAS 2.0	To fill (Score)	Optional	Yes
QOL SF 36	To fill (Score)	Optional	Yes
QOL Bouchard	To fill (Score)	Optional	Yes
IPAQ	To fill (Score)	Optional	Yes
FSS	To fill (Score)	Optional	Yes
Other (specify)	To fill	Optional	Yes
**7.Previous/ongoing treatments**			
**Drugs**			
Pain Relief	Yes/No/NA choice	Optional	Yes
ACE Inhibitors	Yes/No/NA choice	Optional	Yes
Diuretics	Yes/No/NA choice	Optional	Yes
Cardiovascular Drugs	Yes/No/NA choice	Optional	Yes
Insulin or Antidiabetics	Yes/No/NA choice	Optional	Yes
Muscle Relaxants	Yes/No/NA choice	Optional	Yes
Psychoactive Drugs	Yes/No/NA choice	Optional	Yes
Allopurinol:	Yes/No/NA choice	Optional	Yes
Other (specify)	To fill	Optional	Yes
**Special diet**			
Sucrose	Yes/No/NA choice	Optional	Yes
Carbohydrate rich	Yes/No/NA choice	Optional	Yes
Protein rich	Yes/No/NA choice	Optional	Yes
Lipid rich	Yes/No/NA choice	Optional	Yes
Other (specify)	To fill	Optional	Yes
**Supplements**			
B6	Yes/No/NA choice	Optional	Yes
Creatine	Yes/No/NA choice	Optional	Yes
CoQ or Idebenone	Yes/No/NA choice	Optional	Yes
Other vitamins	Yes/No/NA choice	Optional	Yes
BCAA	Yes/No/NA choice	Optional	Yes
Carnitine	Yes/No/NA choice	Optional	Yes
Other (specify)	Yes/No/NA choice	Optional	Yes
**Rehabilitation program**	Yes/No/NA choice	Optional	Yes
**Other treatments (specify)**	To fill	Optional	Yes
**8.Services provided**			
Currently working	Yes/No/NA choice	Optional	Yes
Benefited from specific healthcare	Yes/No/NA choice	Optional	Yes
Changed job because disease	Yes/No/NA choice	Optional	Yes
Employer modified environment because disease	Yes/No/NA choice	Optional	Yes

*Abbreviations*: *ACE* Angiotensin-converting enzyme, *ACTN3* Alpha-actinin-3, *AMPD1* Adenosine monophosphate deaminase 1, *BCAA* Branched chain amino acids, *CK* Creatine kinase, *CoQ* Coenzyme Q, *COPD* Chronic obstructive pulmonary disease, *FSS* Fatigue severity scale, *HR* Heart rate, *IPAQ* International physical activity questionnaire, *PGC* Progastricsin, *QOL SF36* The 36-Item Short Form Health Survey questionnaire, *VO2 peak* Peak oxygen uptake, *WHO DAS 2.0* World Health Organization Disability Assessment Schedule 2.0

^a^ For most variables with “Yes/No (NA) choice”, if answer is “Yes”, new field(s) with additional related questions open (e.g., if “Myoglobinuria = yes”, then an additional field: “Frequency” opens; if “Muscle weakness = yes”, then new fields open asking for affected muscles and severity; if the answer for a specific treatment is “Yes”, new fields open asking for frequency of use and whether it was beneficial or not)

^b^ All data introduced in “Personal Data” section is encrypted and not available for anybody, except for the physician who registered this particular patient

^c^ Demographic data is automatically generated from the data introduced in the section of personal data

### Data collection, storage, and access policy

The EMR complies with the confidentiality policies of the EU and with national directives and regulations on ethical aspects of patients’ data processing.

The EUROMAC project has a public website (http://www.euromacregistry.eu, see below), aimed to promote the knowledge and dissemination of the project. The registry website (https://www.registryeuromac.eu) is hosted separate from the public website, as a secure network. Local databases are the entry points where data is collected, encrypted, pseudo-anonymized, and then transferred to a central registry, which currently stores all the data in a secure and inexpensive system. To reassure the safety of the instrument, a back-up registry is maintained in a participating country (Italy).

The applicative of the EMR has been designed, developed and periodically updated by the Italian GARR Consortium (www.garr.it/b/eng/garr-en/about-us). It includes a public area, in which the informed consent form can be downloaded, and a section for professional registration, which provides a user account to clinicians that enables them to have access to the private area, accessed through a personal username and a password previously provided, where registered clinicians can enter, edit and modify data from their patients. As indicated above, patients’ data are encrypted and pseudo-anonymized for research purposes, but linked to their patient sources through an encrypted algorithm. Following informed consent, data from each patient can be uploaded onto the encrypted web-based registry with a unique code in the database record. The link between the code of a patient and his/her identity can only be known by the physician who got the informed consent from the patient. This identity can be revealed by this physician only under specific conditions clearly indicated in the consent form. The coordinator of the registry has the means to de-anonymize the patients’ codes too, but is only allowed to do it if the specific physician is missing or not able to be contacted.

Registered patients are able to log in with a personal password, review their own information and complete selected sections with their personal experiences. None of the recruited participants or participating doctors is allowed to see data from other parties.

As mentioned above, the EUROMAC project also has its own public webpage that provides news, updates and basic information on the disease and the EMR. It also includes a link to the applicative of the EMR destined to medical doctors and patients. This public webpage has been translated into nine languages (English, German, Spanish, French, Italian, Greek, Danish, Catalan and Turkish), but the registry platform is in English only.

### Expected outcomes

Generation of a database aimed at improving access of patients to specialized care, improving knowledge of the natural history of these disorders and generation of treatment guidelines. The database will facilitate the involvement of the biopharmaceutical industry and governments. Additional outcomes include the dissemination of the EUROMAC website at neurology and neuromuscular meetings, the publication on the EUROMAC website contact details of all European diagnostic labs, together with specialist clinics, and the implementation of training courses and patient support groups meetings.

### Ethics

One of the core WP was dedicated to the ethical and legal issues of the project. Among other elements, this WP included the establishment of an Ethics Committee, nominated at the kick-off meeting, and the generation of an Ethics Report before the Registry was active. The generation of the Ethics Report constituted a mandatory milestone during the development of the project. This document establishes all internal rules and procedures designed to ensure that the project and the Registry meet the mandatory regulations of the EU and all countries to which EUROMAC partners belong. Among the elements contained in the Ethics Report, the following items can be mentioned:

1. Review of the existing legislations in all the countries involved in the registry.
2. Description of the process flow and criteria to determine it.
3. List of the reference EUROMAC investigators for every country (country managers).
4. Patient information and consent form, explaining the registry, data collection, and option to opt-out at any time.

One of the main ethical issues to be considered in patient registries is that data protection is an obvious prerequisite, including transparency of the procedures of data collection and data analyses. Therefore, this was a core part of the Ethics Report, as well as one of the main subjects to be addressed in the WP dedicated to the ethical and legal issues.

The project was approved by the Institutional Review Board of the Coordinating Center (Vall d’Hebron Research Institute, Barcelona), and by review boards of each institution recruiting participants.

### Results

The main outcome of the registry is the scientific information derived from the patient database analysis, and for this we refer to the companion article in this issue to evaluate how the registry can be useful to expand our knowledge about the disorders [21]. However, the generation of the first European registry dedicated to rare muscle glycogenoses can be considered a result in itself. The following summary of achievements may be useful to suggest procedures and tips to be considered when dealing with other similar registries:

1. A dynamic clinical and research network focused on muscle glycogenoses has been established at the European level, allowing better communication and sharing of available information for designing and developing large-scale clinical trials.
2. EUROMAC has significantly contributed to the effective dissemination and raising the awareness of the diseases not only in those countries with EUROMAC partners, but also in countries not initially represented (e.g., Netherlands or Poland). The EUROMAC project website (http://www.euromacregistry.eu) accumulates 9200 visits (December 2019), which is a good indicator of the awareness of the project.
  In the process of the project development, the Dissemination Plan promoted the organization of events, publications and dissemination material (leaflets, brochures, books) in different languages generated and distributed in the frame of EUROMAC.
  Specifically, some of the activities conducted in the frame of the Dissemination Plan were: (i) creation of a EUROMAC project video (https://www.youtube.com/watch?v=NXvmRGLcIy8&list=PLECPX8EZXgfMflBqX4hWLYc4n_4bieuwV), as well as a video about McArdle patients’ experiences (https://www.youtube.com/watch?v=F49Vy8LTkjg&t=11s), both subtitled in several languages (1919 and 1433 visits in all languages respectively, November 2019); (ii) translation of several information booklets on McArdle Disease in several languages; (iii) events and formative meetings (McArdle exercise testing workshop (July 2014, Madrid, Spain) [22], 211th European Neuromuscular Centre International Workshop (April 2015, Naarden, Netherlands) [23], EUROMAC symposium (April 2016, London, UK), McArdle study day (February 2016, Belgrade, Serbia) and EUROMAC teaching course (May 2016, Warsaw, Poland).
3. The registry has been implemented and registering activity started in September 2015. At present (November 2019), 313 patients have been registered, which accomplishes the proposed objective for this project to register 250 patients. These 313 patients represent approximately 10% of the total number of patients in the participating countries, based on prevalence estimates, which constitutes an excellent percentage of entries 4 years after the implementation of the operative registry.
4. EUROMAC has contributed so far to the implementation of at least two clinical trials (ClinicalTrials.gov: NCT03112889 and NCT02432768) [24].
5. Many patients were very happy to hear that the diagnosis made, in most cases, years ago, obtained new research interest, and they were satisfied to be able to contribute to the body of knowledge on their rare disease, and the registry encouraged them to contact and participate in patients organizations.

## Discussion

EMR is an entirely public and secure database designed to contain the medical data from patients with McArdle Disease and related conditions. The EUROMAC network collaboration was initiated in 2012 with the main objective of improving diagnosis and care for people living with these rare disorders, to reduce serious complications and social care costs and to provide a research platform for future clinical trials by raising awareness and increase knowledge with respect to the natural history of these disorders. In patients with these disorders, delayed diagnosis and incorrect medical advice leads to debilitating symptoms and increased risk of life-threatening crises necessitating admission to critical care, all of which can be prevented by correct medical care. The EUROMAC was a complex undertaking that involved multi-disciplinary teams of 18 partners from nine countries. Owing to the low number of patients with rare muscle glycogenolytic disorders at national level, the EUROMAC registry was created at European level establishing collaborative efforts to ensure data collection and its maintenance.

To date, the project fulfilled the expectations for an accessible and readily visible e-platform where the scientific community can count and properly describe most patients with rare muscle glycogenoses. Registration of the patients proceeded steadily once the registry functions were in place, reaching the number of 313 by November 2019. The choice of enriching the registry with a wealth of clinical and functional information makes the registry, in its present form, a good starting point for a dynamic specialized e-health record, but is also the main reason for its underperformance in number of registered patients. Although the initial expectations for registering rate were higher than those actually achieved (the initial target was having 500 registered patients at the end of the CHAFEA-funded period), this number had to be lowered to a more realistic value (250 patients) in one of the interim amendments of the project. Indeed, when the consortium started registering patients, it was soon detected that efficiency in loading the patients’ data was below initial expectations because of the need to obtain a signed consent form from each subject, which was the main rate-limiting factor. Moreover, the richness of the clinical information required, which demanded for direct patient re-examination for most of the requested measures, also added to the delay in registration, which is still on going. This element is confirmed by the unequal speed and number of data entry by the various partners: those who have routine scheduled visits of the patients could easily retrieve the needed data, those performing diagnostic workout but no clinical follow-up could not keep up. Taken together, we assumed that lowering the initial registering activity rate expectations was fully justified if that allowed that the quality of the data (and so the usefulness of the registry) is more robustly ensured.

In addition to the scientific observations and conclusions derived from data analysis, presented in the companion paper [21], when we look to what we learned from the registering activity from a methodological and procedural point of view, we can raise the following conclusions:

- Countries providing centralized care focused on muscle glycogenoses, such as the UK, have shown better rates of registering activity than those countries where management and expertise of these disorders is fragmented across multiple centres. Although this does not make any judgment about whether centralization is a better strategy, it does open the question to find strategies to engage other professionals to participate in EUROMAC, serving as a pan-European reference organization to coordinate and manage these activities.
- The rate-limiting step for the registering activity is obtaining signed informed consent from patients.
- The active role of patients’ organizations is of the utmost importance to maximize the chances to recruit patients for the registry. Countries with more active patients’ organizations minimized the aforementioned bottleneck (informed consent) and helped to engage the participation of more patients.
- Most patients are diagnosed based on a molecular genetic confirmatory test, clearly reflecting the shift in diagnostics toward a molecular genetic approach.
- The quality of recorded data is generally good, but certain variables are not reported in most of the patients frequently enough. This drawback will be overcome in the future by the re-entry of those patients’ information in a retrospective manner.
- The data set offers a unique platform for the EUROMAC consortium and collaborators to work on in the future. The registry is a valuable asset not only for the members of the current EUROMAC consortium, but also for new collaborators that are already contributing and other colleagues who are in the process of arranging their capability to participate.

Additionally, the expected long-term impacts of the EUROMAC project at an international level are: a) improved access of patients to specialised care; b) facilitated involvement of national governments and regulatory agencies; c) improved knowledge of the natural history of McArdle Disease and other rare muscle glycogenoses; d) reduced average delay in diagnosis; e) reduced risk of incorrect advice leading to debilitating symptoms and increased risk of life-threatening crises; and f) extended use of the registry to all other European countries. All these long-term expected impacts will need further analyses after extended time of registering patients.

The project has also contributed to the establishment of a communicative channel with the biopharmaceutical industry, because the registry will improve the understanding of the natural history of these diseases, and the available data enables designing better clinical trials and evaluating potential relevant clinical endpoints.

A positive aspect of the project is the dissemination and the raised awareness about this neglected group of rare diseases. The power of a dedicated multilingual web site and the possibility for patients to directly contribute was hailed by most patients as a very positive novelty. The distribution of multilingual information material on McArdle Disease is a concrete achievement that will continue beyond the project life. Over 90% of the patients contacted for first data collections were happy to be contacted again for follow-up. Participation by consortium partners to all meetings forged a strong sense of belonging among the partners, which functioned as accretion nucleus, leading other European partners to join the consortium (Netherland and Poland). Actually, incorporation of new active EUROMAC partners has been encouraged and welcome by the initial EUROMAC consortium, among those participants that have joint as registering professionals following the mechanism described in the Methods and Results section above (Data collection, storage, and access policy), when they show interest to become active participating partners. The training sessions held in London and Madrid, as well as the workshop held in Naarden [23], achieved the double goal of standardizing assessment procedures and increase knowledge on specific functional and clinical aspects of the diseases. They were attended by a large number of professionals and were rated by participants as very useful and qualitatively excellent. In addition, special efforts dedicated to disseminate our project in Eastern countries, with meetings held in Warsaw and Belgrade, have contributed to increase knowledge and awareness about these disorders in Eastern Europe. We expect that these dissemination strategies will contribute to improve diagnosis of these diseases in this region, where underdiagnose of these diseases seems to be of special concern.

## Conclusions

In conclusion, EMR represents a major step forward in the knowledge of muscle glycogenoses with relevant clinical outcomes at the diagnostic, epidemiologic, clinical and research levels.

## Data Availability

Not applicable.

## Abbreviations

AGSD-UK: Association for Glycogen Storage Disease (UK)
CK: Creatine kinase
EMR: EUROMAC registry
WP: Work package
